# Elevated premature ventricular complex counts on 24-hour electrocardiogram predict incident atrial fibrillation and heart failure—A prospective population-based cohort study

**DOI:** 10.1016/j.hroo.2022.05.008

**Published:** 2022-05-16

**Authors:** Alexandra Måneheim, Gunnar Engström, Tord Juhlin, Anders Persson, Suneela Zaigham, Linda S.B. Johnson

**Affiliations:** ∗ Lund University, Lund, Sweden; † Department of Clinical Sciences, Skåne University Hospital, Malmö, Sweden

**Keywords:** Epidemiology, 24-Hour electrocardiogram, Ventricular extrasystole, Supraventricular extrasystole, Population, Atrial fibrillation, Heart failure, Ambulatory electrocardiogram

## Abstract

**Background:**

Premature ventricular complexes (PVCs) are known to predict heart failure (HF) and premature atrial contractions (PACs) are known to predict atrial fibrillation (AF) and stroke. PVCs and PACs share pathophysiological mechanisms; however, the combined effects of PVCs and PACs on HF, AF, and stroke risk have not been studied.

**Objectives:**

To study elevated PVC counts on 24-hour electrocardiogram monitoring (24hECG) in relation to incidence of AF, HF, and stroke, and whether this effect is altered by PAC frequency.

**Methods:**

The prospective population-based Malmö Diet and Cancer study includes 24hECG registrations in 375 AF- and HF-free subjects (mean age 65 years, 55% women). During 17 years of follow-up there were 28 HF, 89 AF, and 28 stroke events. The hazard ratios (HR) of elevated PVC counts (defined as the top quartile, ≥77/24 hours) vs lower quartiles were assessed using multivariable adjusted Cox regression models.

**Results:**

Elevated PVC counts predicted incident AF (HR 1.9, 95% confidence interval [CI] 1.2–3.0) and HF (HR 3.1, 95% CI 1.4–7.0). Results were similar after adjustment for NT-proBNP and PACs. Multiform PVCs were associated with even higher risks (HR 2.8, 95% CI: 1.7–4.6 for AF; HR 5.0, 95% CI 2.2–11.7 for HF), as was the presence of both elevated PACs and PVCs (9% of the population, HR 4.1, 95% CI 2.4–6.8 for AF and HR 4.3, 95% CI 1.7–11.4 for HF). No significant association was found between elevated PVC counts and incident stroke.

**Conclusion:**

Elevated PVC counts predict incident AF and HF, particularly if PVCs are multiform or occur in combination with elevated PAC counts.

**Key Findings**

▪ Premature ventricular complexes (PVCs) are independently associated with incident atrial fibrillation and heart failure in a population-based cohort.
▪ This association is mainly driven by multiform PVCs.
▪ A combination of both PVCs and premature atrial contractions further increases the risk of incident atrial fibrillation and/or heart failure.

## Introduction

Premature ventricular complexes (PVCs) are commonly found on ambulatory electrocardiogram (ECG) recordings and are associated with increasing age and height, male sex, and modifiable risk factors such as hypertension,^1^, ^2^, ^3^ smoking, and low physical activity.^2^^,^^3^ Pathophysiological mechanisms and triggers for PVCs are to some extent shared with premature atrial complexes (PACs).^4^, ^5^, ^6^, ^7^, ^8^, ^9^, ^10^, ^11^ The latter can predict atrial fibrillation (AF) and ischemic stroke, even at relatively low levels.^12^^,^^13^

It is well established that high PVC burdens can cause cardiomyopathy^4^^,^^14^^,^^15^; however, there is limited knowledge regarding the potential relationship between lower PVC burdens and risk of incident heart failure (HF), as well as AF and stroke. To our knowledge, only 1 previous population-based study has investigated the relationship between mildly elevated PVC counts on 24-hour electrocardiogram monitoring (24hECG and incident HF, and previous studies concerning PVCs and risk of incident AF and stroke have not accounted for the levels of PACs on 24hECG.^16^, ^17^, ^18^, ^19^

A combination of PVCs and PACs could indicate a more widespread underlying pathophysiology, affecting both the atria and ventricles. It is not known what independent effects PAC and PVC frequencies could have on incident AF, HF, and stroke. Furthermore, it is not known whether this potential relationship between PVCs, PACs, and incident AF, HF, and stroke could be explained by subclinical cardiac strain, measured by N-terminal pro-B-type natriuretic peptide (NT-proBNP).

The primary aim of the present study is to determine whether PVCs at 24ECG are associated with incident AF, HF, and ischemic stroke. A secondary aim is to study the combined effects of PVCs and PACs on incidence of AF, HF, and stroke.

## Methods

### Study population

**Figure 1:**
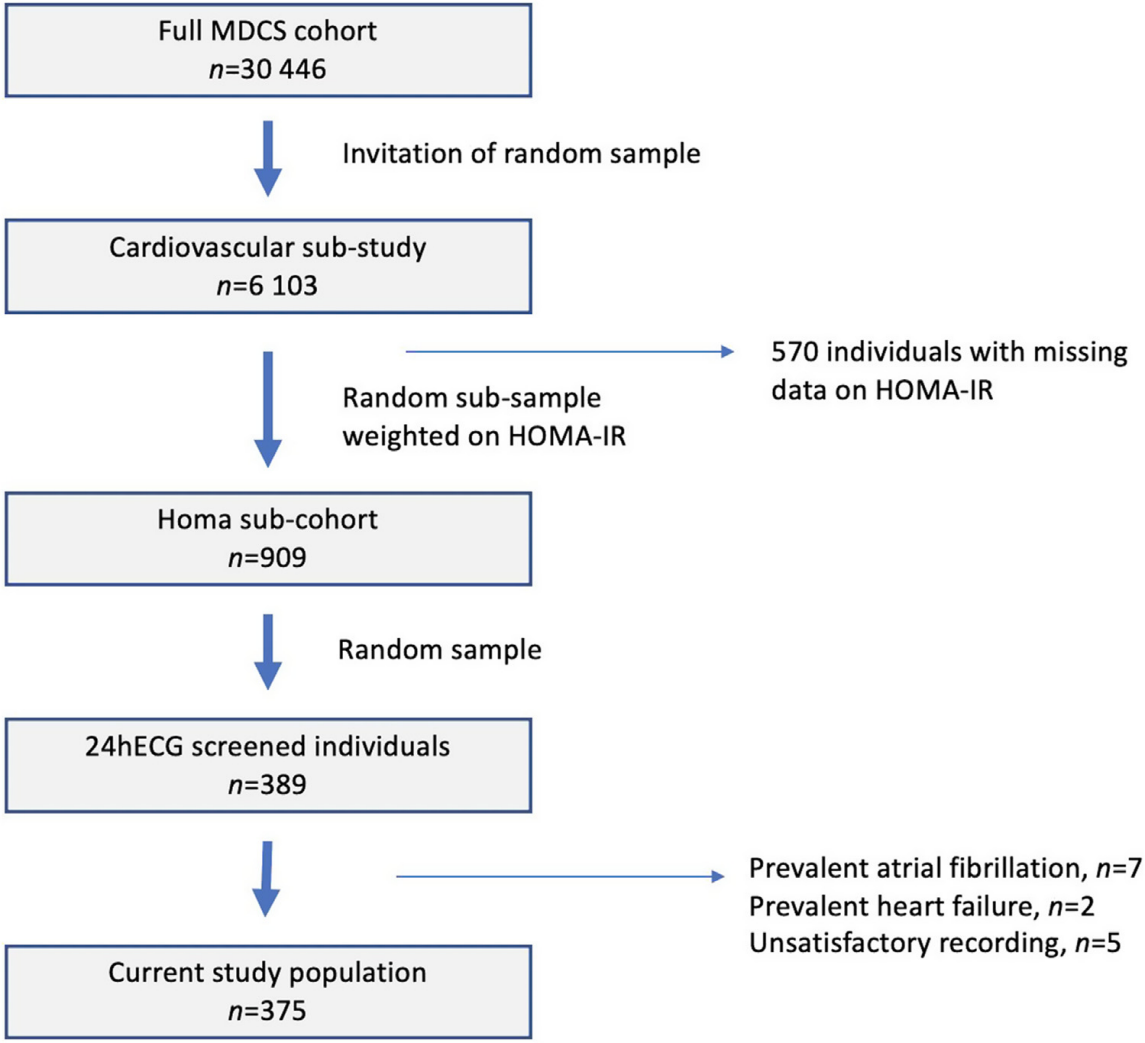
Derivation of the study population. HOMA-IR = homeostatic model assessment of insulin resistance; MDCS = Malmö Diet and Cancer Study; 24hECG = 24-hour electrocardiogram monitoring.

The Malmö Diet and Cancer Study (MDCS) cohort was recruited in 1991–1996 and has previously been described in detail elsewhere.^12^^,^^20^ In brief, all men born between 1923 and 1945 and all women born between 1923 and 1950 who lived in Malmö, Sweden, were invited to participate in this large prospective cohort study. The participation rate was ≈40% and 30,446 individuals underwent a baseline examination consisting of a physical examination, which included weight, height, and blood pressure measurements; an extensive health and lifestyle questionnaire; and fasting blood sampling. All participants provided written informed consent. A random sample from the MDCS cohort, who were examined between 1991 and 1994 (n = 6103), were included in the MDCS cardiovascular substudy. Within this substudy a random sample, stratified by homeostatic model assessment for insulin resistance (HOMA-IR), was invited for reinvestigation in 1999–2000. Higher HOMA-IR was slightly oversampled (15% from quartile 1, 15% from quartile 2, 30% from quartile 3, and 40% from quartile 4). In this population subset 909 individuals underwent more extensive screening, which in a random subsample of 389 individuals included a 24hECG registration (Figure 1).

After exclusion of individuals with prevalent HF (n = 2) prevalent AF (n = 7), and insufficient ECG recording quality (n = 5), a total of 375 individuals (169 men and 206 women) were included in the present study population (Figure 1). For stroke analysis, 3 additional individuals with prevalent stroke at baseline were excluded.

### Data collection

Height and weight were measured standing in light indoor clothes, without shoes. Body mass index was calculated as kg/m^2^. Blood pressure (mm Hg) was measured after 10 minutes of supine rest. Blood samples were drawn after an overnight fast and NT-proBNP was analyzed at the Clinical Biomarkers facility (Science for Life Laboratory, Uppsala University, Uppsala, Sweden). Information regarding smoking status, physical activity, alcohol intake, and current medications was obtained from a self-administered questionnaire at baseline.

The 24hECGs were recorded with X,Y,Z coupling and 256-Hz sampling rate, using a LifeCard CF digital Holter recorder with 12-bit resolution. The 24hECG results were analyzed with the Pathfinder SL analysis tool (Spacelabs Healthcare, Issaquah, WA) and all arrhythmias were visually confirmed. The level of prematurity required for a PAC or PVC classification was at ≥20% shorter than the previous normal R-R interval. A manual classification of PVC forms was conducted by the authors (AM and LSJ). PVCs were classified as uniform when ≥90% were of the same PVC morphology while the multiform PVC group included subjects with 2 or more PVC morphologies comprising at least 10% of the total number of PVCs.

The endpoints were clinical AF (diagnosis codes 427D for ICD-9 and I48 for ICD-10), HF (diagnosis codes 428 for ICD-9 and I11.0 and I50 for ICD-10), or ischemic stroke (diagnosis codes 434 for ICD-9 and I63 for ICD-10), diagnosed in a hospital inpatient or outpatient setting. Subjects were followed until first incident AF, HF, or stroke, respectively, until death or censoring by emigration from Sweden (n = 3). Cases were retrieved from the Swedish National Hospital Discharge Register, which is administered by the Swedish National Board of Health and Welfare. The in-patient register has been in use in the south of Sweden since 1970 and the register became nationwide in 1987. The outpatient register has been maintained since 2000. The validity in this register has been studied and found to be good, and owing to the comprehensive nature of the registries no missing data were introduced at registry linkage.^21^^,^^22^

The study conforms to the Declaration of Helsinki and the regional ethics review board in Lund has approved the study. The last follow-up date for this study was December 31, 2018. Data collection and endpoint retrieval have previously been more thoroughly described elsewhere.^12^

### Statistics

All data were analyzed using Stata version 14.2 (Stata Corporation, College Station, TX). Continuous variables were visually assessed for normality; skewed variables (NT-proBNP) were transformed with the natural logarithm. PVCs and PACs were stratified into quartiles, and the highest quartile was considered elevated. The relationship between PVCs and PACs, as well as between PVCs and NT-proBNP, was assessed using Spearman’s rank correlation coefficient.

The associations between elevated PVCs and incident AF, HF, and stroke, respectively, were analyzed using multivariable Cox regression with age as time scale. Analyses were weighted for the probability of invitation to rescreening based on HOMA-IR measured at the MDCS cardiovascular substudy examination. The validity of proportional hazards assumption was confirmed visually using Nelson-Aalen cumulative hazard plots. Results are reported unadjusted and after multivariable adjustment for sex, smoking status, body mass index, and the use of antihypertensive medication (beta blockers, calcium blockers, or diuretics). To assess the effect of NT-proBNP and PAC frequency, we conducted further subanalyses where log-transformed NT-proBNP and frequent PACs were included. Owing to a few missing data for NT-proBNP, these analyses included 372 individuals in AF and HF analyses and 368 individuals in stroke analyses.

We did several sensitivity analyses. First, a sensitivity analysis that assessed the potential effect of alcohol intake and low physical activity was performed, where these variables were added to the multivariable model (owing to missing data, 373 individuals in AF and HF analyses and 370 individuals in stroke analyses). Another sensitivity analysis concerning coronary artery disease (CAD) was done where individuals with CAD at baseline were excluded (n = 2) and subjects were censored at the time of a diagnosis of incident CAD. In order to determine whether associations between elevated PVCs and the outcomes were driven by extreme values of PVCs, we performed a sensitivity analysis where individuals with PVCs >95th percentile were excluded, and we also assessed the association between PVCs as a continuous variable (transformed with natural logarithm owing to skewness of data) and incident outcome events.

The effect of multiform PVCs was assessed in a multivariable adjusted analysis where subjects with elevated PVC counts were stratified according to the presence of either uniform or multiform PVCs, and subjects without elevated PVC counts were considered the reference category.

## Results

Baseline characteristics are reported in Table 1. Mean age at baseline was 65 (± 6) years, and 55% of subjects were women. The median duration of 24hECG monitoring was 23.5 hours, and the median PVC and PAC counts per 24 hours were 10 (interquartile range [IQR] 2–77) and 39 (IQR 14–132), respectively. Individuals in the top quartile of PVCs were older, were more often men, and had higher systolic blood pressure and a higher NT-proBNP compared to the rest of the study population (Table 1). The median PVC count in the top quartile of PVCs was 279 PVCs/24 hours (IQR 144–870). Among 94 individuals with elevated PVC counts, 48 had uniform PVCs and 46 had multiform PVCs.

**Table 1:** Baseline characteristics by quartiles of premature ventricular complex frequency

	All	Quartile 1	Quartile 2	Quartile 3	Quartile 4	*P* value
No of individuals	375	86	101	94	94	
Range of PVCs/24 hours	0–19,990	0–2.1	2.1–10.0	10.0–77.2	77.2–19,990	
Age at baseline, years	64.5 ± 5.9	62.0 ± 6.4	64.6 ± 5.8	65.2 ± 5.5	66.0 ± 5.0	<.001
Male sex (%)	45.1	36.0	36.0	54.7	53.2	.010
Height, cm	169.0 ± 9.4	168 ± 8.8	167.4 ± 9.9	169.4 ± 9.4	170.5 ± 9.1	.100
Weight kg	77.3 ± 13.3	75.8 ± 13.2	76.5 ± 12.0	77.0 ± 12.8	79.7 ± 14.9	.195
BMI, kg/m^2^	27.0 ± 3.9	26.7 ± 3.9	27.3 ± 4.1	26.8 ± 3.8	27.3 ± 4.0	.601
Systolic blood pressure, mm Hg	143.7 ± 18.5	141.7 ± 19.8	140.2 ± 15.4	144.5 ± 18.5	148.4 ± 19.6	.011
Blood pressure medication, (%)	16.0	15.1	16.8	14.9	17.0	.967
Diabetes (%)	9.3	11.7	9.9	9.2	10.6	.903
Prevalent CAD (%)	1.1	2.3	0	1.1	1.1	.497
High physical activity† (%)	25.2	19.8	22.2	31.6	26.9	.343
Low physical activity‡ (%)	24.9	25.6	31.3	21.1	21.5	.356
Ever smoker (%)	58.7	60.0	59.0	53.7	61.7	.647
Current smoker (%)	22.7	20.9	31.0	16.8	21.3	.129
Alcohol, g/day, median (IQR)	7.0 (1.3–14.3)	6.1 (1.3–13.0)	6.5 (0.9–14.1)	7.0 (1.3–14.0)	8.8 (2.8–17.7)	.407§
HOMA-IR, median (IQR)	2.1 (1.4–3.1)	2.1 (1.3–3.2)	2.2 (1.5–3.3)	2.2 (1.6–3.2)	1.7 (1.2–3.0)	.241§
NT-proBNP, pg/mL, median (IQR)	10.8 (1.7-32.7)	3.7 (0.5-28.0)	11.3 (1.8-32.1)	13.4 (4.2-24.8)	15.5 (4.1-62.9)	.004§

Continuous variables are reported as mean ± standard deviation except for alcohol use, HOMA-IR, and NT-proBNP, which are reported as median (IQR).

*P* values are calculated using χ^2^ test for binary variables or ANOVA for continuous variables.

BMI = body mass index; CAD = coronary artery disease (history of myocardial infarction or coronary artery bypass graft); HOMA-IR = homeostatic model assessment of insulin resistance; IQR = interquartile range; PAC = premature atrial complex; PVC = premature ventricular complex.

† Upper quartile of physical activity score.

‡ Lower quartile of physical activity score.

§ Variable is log-transformed prior to ANOVA testing owing to skewness in distribution.

Three individuals had no PACs and 43 individuals had no PVCs. Only 2 individuals had neither PACs nor PVCs. PACs and PVCs were moderately positively correlated, Spearman’s rho 0.31 (*P* < .0001). There was also a statistically significant but weak correlation between PVCs and NT-proBNP, Spearman’s rho 0.19 (*P* = .0002)*.*

### Incident AF, HF, and stroke in subjects with elevated PVC counts

Median follow-up time was 17 years, during which there were 89 incident AF events (cumulative incidence 24%), 28 incident HF events (cumulative incidence 8%), and 28 incident ischemic stroke events (cumulative incidence 8%). Ninety individuals died during follow-up. Incidence rates by quartiles of PVCs in men and women are presented in Table 2. An elevated PVC count was independently associated with an almost doubled risk of incident AF (hazard ratio [HR] 1.9, 95% CI 1.2–3.0, *P* = .004) and 3-fold risk of incident HF (HR 3.1, 95% CI 1.4–7.0, *P* = .007), compared to lower quartiles of PVCs. There were no statistically significant associations between elevated PVC counts and incident ischemic stroke. Unadjusted and multivariable adjusted Cox regression models are presented in Table 3. Moreover, elevated PVCs were not significantly associated with death (HR 1.5, 95% CI 0.9–2.4, *P* = .08).

**Table 2:** Incidence of atrial fibrillation, heart failure, and stroke by quartiles of premature ventricular complex frequency

	All		Quartile 1 0–2.1 PVCs/24 hours		Quartile 2 2.1–10.0 PVCs/24 hours		Quartile 3 10.0–77.2 PVCs/24 hours		Quartile 4 77.2–19,990.5 PVCs/24 hours	
	Incidence/1000 person-years (95% CI)	Events/subjects	Incidence/1000 person-years (95% CI)	Events/subjects	Incidence/1000 person-years (95% CI)	Events/subjects	Incidence/1000 person-years (95% CI)	Events/subjects	Incidence/1000 person-years (95% CI)	Events/subjects
Atrial fibrillation	16.4 (13.3–20.2)	89/375	11.9 (7.2–19.7)	15/86	10.1 (6.1–16.7)	15/101	17.8 (12.0–26.3)	25/94	26.9 (19.2–37.6)	34/94
Heart failure	4.9 (3.4–7.1)	28/375	3.0 (1.1–7.9)	4/86	3.2 (1.3–7.8)	5/101	3.3 (1.4–8.0)	5/94	10.3 (6.1–17.3)	14/94
Ischemic stroke	4.9 (3.4–7.2)	28/372	1.5 (0.4–6.0)	2/85	4.5 (2.2–9.5)	7/101	6.2 (3.2–11.8)	9/94	7.6 (4.1–14.1)	10/92

CI = confidence interval; PVCs = premature ventricular complexes.

**Table 3:** Cox regression models for incident atrial fibrillation, heart failure and stroke for the top quartile of premature ventricular complex frequency

Top quartile of PVC/h (n = 94)						
	Atrial fibrillation		Heart failure		Stroke	
	HR (95% CI)	*P* value	HR (95% CI)	*P* value	HR (95% CI)	*P* value
Unadjusted	2.1 (1.4–3.3)	.001	3.6 (1.7–7.7)	.001	1.9 (0.9–4.1)	.119
Multivariable adjusted†	1.9 (1.2–3.0)	.004	3.1 (1.4–7.0)	.007	1.7 0.8–3.9)	.190

All models are weighted for the stratified sampling. Age was used as time scale.

Reference category: lower quartiles (1–3) of PVCs.

CI = confidence interval; HR = hazard ratio; PVC = premature ventricular complex.

† Adjusted for sex, ever-smoking status, body mass index, and the use of blood pressure medication.

### Subanalyses and sensitivity analyses

The inclusion of NT-proBNP to the multivariable adjusted model did not alter results substantially, nor did the further inclusion of elevated PAC counts (HR 1.9, 95% CI 1.2–2.8; *P* = .005 for AF and HR 2.9, 95% CI 1.3–6.5; *P* = .008 for HF). When height, alcohol intake, and low physical activity were added to the multivariable model, results for AF were unchanged and for HF slightly attenuated (HR 1.9, 95% CI 1.2–3.0, *P* = .005 for AF and HR 2.7, 95% CI 1.1–6.5, *P* = .025 for HF). There was no evidence of confounding or mediation by prevalent or incident CAD. In a subanalysis in which subjects with prevalent CAD were excluded and with censoring at the time of an incident CAD diagnosis, results for top quartile of PVCs were largely unchanged (multivariable adjusted HR 1.8, 95% CI 1.1–2.9; *P* = .015 for AF and HR 3.6, 95% CI 1.4–9.0; *P* = .007 for HF).

Finally, in order to assess whether the association between elevated PVC counts and incident AF and HF was driven by subjects with the highest daily PVC counts, we performed a subanalysis where all subjects with PVCs above the 95th percentile (>1043 PVCs/24 hours, n = 19) were excluded. After multivariable adjustment, results were substantially unchanged (HR 1.8, 95% CI 1.1–3.0, *P* = .019 for AF and HR 3.0, 95% CI 1.3–7.2, *P* = .011 for HF). When PVCs were considered as a continuous variable, the associations between PVCs and AF and HF remained significant (HR 1.2, 95% CI 1.0–1.4, *P* = .014 for AF and HR 1.2, 95% CI 1.0–1.5, *P* = .042).

### Incident AF and HF in subjects with elevated PAC and PVC counts

We also assessed whether the combination of PVCs and PACs was associated with a higher risk of developing incident AF or HF. The highest risks of AF and HF were found among subjects with both elevated PVC and PAC counts (n = 34), among whom the risks AF and HF were quadrupled compared to subjects with low levels of PVCs and PACs (Table 4).

**Table 4:** Multivariable Cox regression models for atrial fibrillation, heart failure, and stroke by strata of premature ventricular complexes and premature atrial complexes

	Low PVCs and PACs		High PVCs, low PACs		High PACs, low PVCs		High PVCs and PACs	
	HR (95% CI)	Events/subjects	HR (95% CI), *P value*	Events/subjects	HR (95% CI), *P value*	Events/subjects	HR (95% CI), *P value*	Events/subjects
Atrial fibrillation	ref	34/221	1.3 (0.7–2.5), *.455*	13/60	1.5 (0.8–2.7), *.200*	21/60	4.1 (2.4–6.8), *<.001*	21/34
Heart failure	ref	10/221	2.1 (0.7–6.4), *.196*	5/60	1.0 (0.3–3.7), *.998*	4/60	4.3 (1.7–11.4), *.003*	9/34
Stroke	ref	8/220	2.3 (0.7–7.5), *.183*	5/59	2.6 (0.9–7.5), *.070*	10/60	3.1 (1.0–9.7), *.055*	5/33

All models are weighted for the stratified sampling. Age was used as a time scale. Adjusted for sex, ever-smoking status, body mass index, and the use of blood pressure medication.

Low PVCs and PACs = quartiles 1–3 for PVCs and PACs; high PVCs and PACs = quartile 4 for PVCs and PACs.

CI = confidence interval; HR = hazard ratio; PAC = premature atrial complex; PVC = premature ventricular complex.

### Incident AF and HF by uniform and multiform PVCs

Compared to subjects without frequent PVCs, those with an elevated PVC count and multiform PVCs were at an almost 3-fold risk of incident AF, while the risk associated with an elevated PVC count with uniform PVCs was nonsignificant (HR 2.8, 95% CI 1.7–4.6; *P* < .001 for multiform PVCs and HR 1.2, 95% CI 0.6–2.2; *P* = .646 for uniform PVCs, after multivariable adjustment). Multiform PVCs were also highly associated with incident HF while uniform PVCs were not (HR 5.0; 95% CI 2.2–11.7; *P* < .001 for multiform and HR 0.9, 95% CI 0.2–4.2; *P* = .844 for uniform), compared to subjects without frequent PVCs and with multivariable adjustment.

## Discussion

Elevated PVC counts at 24hECG independently predict incident AF and HF in a general population sample, even at levels that would be considered low in a clinical setting. This association was independent of PACs, which were weakly to moderately correlated with PVCs. The combination of elevated PVCs and PACs was associated with the highest increase in risk, and one can hypothesize that this combination may reflect a more widespread underlying cardiac pathophysiology*.* Interestingly, it seems like multiform PVCs are the main contributors to the increased risks of developing AF and/or HF, which also supports this idea. The association between PVCs and incident AF and HF, respectively, was not affected by adjustment for NT-proBNP, nor by adjustment for frequent PACs.

Unlike other previous studies, we found no statistically significant association between elevated PVC counts and incident stroke. The nonsignificant associations were consistently positive, however, which may indicate that the lack of statistically significant association could be due to the relatively small sample size.

The 24hECG is a relatively nonexpensive, noninvasive, and widely available diagnostic method, and for these reasons it is in widespread clinical use. Elevated PVC counts constitute a common finding, which currently often does not lead to any clinical interventions. The present study implies that elevated PVC counts, even at low levels, may be used to identify subjects at high risk of HF and AF, especially if PVCs are multiform or if a subject presents with both elevated PVC and PAC counts. There is a potential that preventive measures, such as lifestyle intervention and repeated clinical examinations to detect AF before the occurrence of stroke, could lead to improved outcomes. Future studies that address potential drug therapies that have the potential to reduce AF and HF risk in patients with elevated PAC and PVC counts would be of interest, for example sodium-glucose cotransporter-2 inhibitors as a preferred treatment for diabetic patients with PVCs.^23^

A few previous studies have reported PVCs as a risk factor for developing AF and/or HF based on 24hECG examinations, notably the Cardiovascular Health Study, which found a 3-fold increase in HF risk associated with a top-quartile PVC count in an elderly general population sample.^17^^,^^24^ Our findings are thus in concordance with the Cardiovascular Health Study, and extend upon previous studies by being the first to study the interaction between PACs and PVCs on 24hECG, as well as to adjust for NT-proBNP. PACs and PVCs share risk factors, and NT-proBNP is a well-known predictor of both AF and HF. The European Heart Rhythm Association has recently recommended that individuals with PVC counts >500 PVC/24 hours should be referred for further electrophysiological and functional heart screening.^25^ Our study indicates that levels of PVCs previously considered clinically insignificant are in fact predictors of future AF and HF events in the general population.

It is not clear whether high PVC counts at baseline are causally related to the risk of incident AF and/or HF. Possibly, PVCs leading to ventricular dyssynchrony and left ventricular dysfunction, as well as atrial contractions occurring without subsequent opening of the atrioventricular valves, could increase atrial pressure and stretch, which in turn could promote structural changes in the atria. Hypothetically, retrograde ventriculoatrial activation could also act as a PAC and trigger AF. However, since increased risk of both AF and HF was found even at a modestly increased frequency of PVCs corresponding to a low percentage of PVCs to the total number of beats, we consider it more likely that PVCs are a marker for overall cardiac pathology affecting the electrophysiological properties of the heart muscle.

### Strengths and limitations

Even though the association between elevated PVC counts and incident AF and HF, respectively, is robust, sample size constraints do not allow us to examine the optimal threshold above which PVC counts should be an indication for further examinations. The PVC count in the top quartile, in this cohort of individuals in the general population, is in a clinical context rather low. A study with a larger sample size as well as studies of daily PVC variability could possibly find such threshold.^26^ Furthermore, the natural course of PVC frequency over time is not known, and we do not know whether the study participants in this cohort had higher or lower PVC counts during follow-up. Future studies that address the natural history of PVC frequency and risk factors for PVC progression would be of interest.

Our sample of individuals with a combination of elevated PVC and PAC counts was small. However, the confidence intervals surrounding the markedly elevated HRs were well above 1, and results were consistent for both AF and HF, as well as across models. For this reason, we think that the likelihood that this represents a chance finding is low. Nevertheless, residual confounding is possible, and the findings of this study will need to be confirmed in other populations.

A limitation of this study is that we did not have echocardiography as part of the study, and we therefore cannot know whether subjects had preserved or reduced ejection fraction at baseline. Additionally, we have no information regarding the subtypes of incident HF (with preserved or reduced ejection fraction). Nevertheless, all cases of HF were clinical cases diagnosed in a hospital setting, and not incidental findings, and we have also done a subanalysis where we adjust for NT-proBNP at baseline.

## Conclusion

Elevated PVC counts on 24hECG predict incident AF and HF, even at rather low levels. The association is independent of several clinical risk factors for AF and HF, as well as NT-proBNP and PAC counts; however, the risk is highest in subjects with multiform PVCs or with a combination of PVCs and PACs.
